# Evaluation des indicateurs d’alerte précoce de la résistance du VIH aux ARV en Côte d’Ivoire en 2011

**DOI:** 10.11604/pamj.2016.25.52.6283

**Published:** 2016-09-30

**Authors:** Kouadio Jean Yao, Néto Florence Damey, Diby Jean Paul Konan, Joseph Aka, Sandrine Aka-Konan, Alex Ani, Marguerite Te Bonle, Dinard Kouassi

**Affiliations:** 1 Institut National de Santé Publique, Côte d’Ivoire; 2 Direction de l’'Information, de la Planification et de l’'Evaluation, Côte d’Ivoire; 3 Unité de Formation et de Recherche des Sciences Médicales, Université FHB, Côte d’Ivoire; 4 Programme National de Prise en Charge Médicale des Personnes vivant avec le VIH/sida, Côte d’Ivoire; 5 Unité de Soin Ambulatoire et de Conseil, Abidjan, Côte d’Ivoire; 6 Unité de Formation et de Recherche des Sciences Pharmaceutiques et Biologiques, Université FHB, Côte d’Ivoire

**Keywords:** Indicateur d´alerte précoce, VIH, résistance, Côte d´Ivoire, Early warning indicator, HIV, resistance, Ivory Coast

## Abstract

**Introduction:**

En 2001, l'Organisation des Nations Unies recommandait de rendre disponible les médicaments antirétroviraux dans les pays à ressources limitées. Cependant, l'utilisation de ces médicaments à grande échelle s'accompagne du développement de résistance du virus. En Côte d'Ivoire, plusieurs sites prescrivent les antirétroviraux. Cette étude avait pour objectif d'évaluer les facteurs programmatiques associés à un risque élevé d'émergence de résistance du VIH aux antirétroviraux.

**Méthodes:**

Il s'agit d'une cohorte rétrospective sur 20 sites de prise en charge des personnes vivant avec le VIH. La population d'étude était constituée des personnes ayant initié leur traitement antirétroviral sur les sites en 2008-2009. L'estimation de la taille de l'échantillon a été faite à partir de la stratégie d'échantillonnage de l'OMS.

**Résultats:**

Sur 20 sites, 98% des prescriptions initiales étaient conformes aux directives nationales et 20% des sites avaient 100% de prescriptions conformes. Au total, 33% des patients étaient perdus de vue au cours des 12 premiers mois de traitement antirétroviral et 20% des sites avaient moins de 20% de perdus de vue. A 12 mois, 51% des patients demeuraient sous traitement de première intention approprié et 11% des sites ont atteint le seuil d'au moins 70% de patients sous traitement de première intention approprié. Un seul site n'a pas connu de rupture d'antirétroviraux sur les 12 mois.

**Conclusion:**

Des insuffisances relevées dans la prise en charge des personnes vivant avec le VIH traduisent l'existence d'un risque important de résistance du virus aux antirétroviraux en 2008-2009. Pour minimiser ce risque les pratiques de prescription devraient être améliorées, un système de recherche des absents aux rendez-vous devrait être mis en place et la disponibilité constante des antirétroviraux devraient être assurée.

## Abstract (en)

**Introduction:**

In 2001, the United Nations recommended that antiretroviral (ARV) drugs be made available in resource-limited countries. However, the use of these large-scale drugs is associated with the development of drug-resistant virus. In Ivory Coast, several health care/treatment centres prescribe antiretroviral drugs. This study aimed to evaluate the programmatic factors associated with high risk of emergence of HIV ARV drug resistance.

**Methods:**

We conducted a retrospective cohort study involving 20 health care/treatment centres for people living with HIV. The study population consisted of patients who started HIV treatment at the health care/treatment centres in 2008-2009. Sample size calculation was based on WHO sampling method.

**Results:**

Of the 20 health care/treatment centres, 98% of initial prescriptions were in accordance with national guidelines and 20% of health care/treatment centres had 100% of compliant prescriptions. In total, 33% of patients were lost to follow-up during the first 12 months of antiretroviral therapy and 20% of health care/treatment centres had less than 20% of patients lost to follow-up. At 12 months, 51% of patients were under appropriate first-line treatment and 11% of the health care/treatment centres reached the threshold of at least 70% of patients under appropriate first-line treatment. Only one health care/treatment centre didn't experienced an interruption in antiretroviral therapy over 12 months.

**Conclusion:**

Shortcomings in the treatment of people living with HIV justify the existence of a significant risk of viral resistance to antiretroviral drugs in 2008-2009. In order to minimize this risk prescribing practices should be improved, appointment reminder systems should be implemented and a constant availability of antiretroviral drugs should be ensured.

## Introduction

A l'issue de la Session Spéciale de l'Assemblée Générale des Nations Unies sur le virus de l'immunodéficience humaine/syndrome d'immunodéficience acquise (VIH/sida) en 2001, la recommandation a été faite de rendre disponibles les médicaments antirétroviraux (ARV) dans les pays à ressources limitées. Ceci devrait permettre de réduire la disparité entre pays riches et pays pauvres en matière d'accès aux ARV [1]. L'Organisation Mondiale de la Santé (OMS), suite à cette recommandation, a élaboré des directives pour appuyer et faciliter l'utilisation des ARV dans ces pays. Les composantes clés de ces directives sont : i) le développement de programmes de traitement ARV pour répondre aux besoins des personnes vivant avec le virus de l'immunodéficience humaine (PVVIH) dans les pays à ressources limitées; ii) la standardisation et la simplification des schémas thérapeutiques ARV pour une mise en œuvre efficace des programmes de traitement; iii) l'appui des programmes de traitement par les antirétroviraux (TARV) sur les données scientifiques les meilleures pour éviter le recours à des traitements en dessous des standards qui donnent de mauvais résultats entraînant des résistances parmi les patients [2, 3]. En vue de maitriser cette résistance, l'OMS recommande aux pays de mettre en place une stratégie de prévention et d'évaluation de la résistance aux ARV. Cette stratégie devrait permettre de rendre les programmes de traitement plus performants et minimiser l´émergence des résistances aux ARV. En effet, ces dernières peuvent être prévenues en vue de conserver l´efficacité des schémas thérapeutiques de première et de deuxième intention [4]. La Côte d'Ivoire est l'un des pays les plus touchés par la pandémie du VIH en Afrique de l'ouest [5, 6]. D'après l'Enquête Démographique et de Santé et à Indicateurs Multiples (EDS-MICS) de Côte d'Ivoire 2011-2012, la prévalence du VIH était de 3.7% dans la population générale des sujets de 15-49 en 2011[5]. L'introduction de la thérapie antirétrovirale remonte en 1998 par l'initiative d'accès aux ARV et de l'initiative « 3 by 5 ». Une décennie plus tard, le nombre de personnes vivant avec le PVVIH/SIDA bénéficiant du traitement par les ARV ne cesse de croître ; il est passé de 2100 en 2003 à environ 93 065 en 2011 et 109 525 en 2012 [7]. En 2010, le pays s'est doté d'un plan de surveillance de la pharmacorésistance du VIH couvrant la période 2010-2014. Une étude a été réalisée pour évaluer les indicateurs d'alerte précoce (IAP) de la résistance du VIH aux ARV. Cette étude avait pour but de contribuer à l'amélioration des prestations dans les structures de prise en charge des personnes vivant avec le VIH afin de minimiser la survenue des résistances du VIH aux ARV. L'objectif était d'évaluer les facteurs programmatiques pouvant être associés à un risque élevé d'émergence de résistance du VIH aux ARV. De façon plus spécifique il s'agissait de : (i) déterminer les pratiques de prescription des ARV dans les sites de PEC ; (ii) déterminer la proportion de perdus de vue au cours de la première année de traitement ARV ; (iii) déterminer la proportion de patients sous régime thérapeutique de première ligne 12 mois après le début du traitement ARV ; (iv) déterminer la régularité de l'approvisionnement des sites en ARV pendant une année de traitement.

## Méthodes

Il s'agissait d'une cohorte rétrospective. L'étude a porté sur 20 sites sélectionnés après une analyse situationnelle conduite dans 30 structures sanitaires de dispensation des ARV reparties dans cinq régions sanitaires du pays. Le choix des sites de ces régions s'est fait tenant compte à la fois de leur accessibilité et du plan de progression visant à couvrir l'ensemble du territoire national, conformément au plan national de surveillance de la pharmacorésistance du VIH. Comme critères de sélection, les sites devaient être ouverts au moins depuis le 1er janvier 2008, être toujours fonctionnels, disposer de données permettant de renseigner les 4 IAP retenus et n'avoir pas interrompu les activités de prise en charge (PEC) sur le site. La population de l'étude était constituée des dossiers de patients vivant avec le VIH (adultes ou enfants) ayant initié un traitement ARV entre le 1er mars 2008 et le 28 février 2009. La durée de suivi des PVVIH étaient de 15 mois, à compter de la date de mise sous TARV. Ont été exclus de l'étude, les dossiers de patients qui ne portaient pas de numéro d'identifiant unique, de date du début du TARV, de schéma thérapeutique initial. Pour les enfants, ont été exclus les dossiers ne précisant pas la dose quotidienne des ARV, le poids et l'âge. Pour chaque site, la taille de l'échantillon a été estimée à partir de la stratégie d'échantillonnage de l'OMS sur les IAP, avec une table qui donne la taille de l'échantillon en fonction du nombre annuel de patients ayant initié un traitement [4]. Une fois sur le site, les dossiers des patients ayant initié le traitement pendant la période concernée étaient identifiés. La taille des échantillons était déterminée et un tirage aléatoire systématique a permis de repérer les dossiers à enquêter. Les données collectées étaient d'une part des données générales permettant d'identifier la structure, la taille requise pour l'échantillon et le nombre effectif de patients enrôlés, d'autre part des données spécifiques pour le calcul de chaque IAP. Les sources de données étaient constituées des dossiers individuels des patients sous ARV, des registres de suivi des patients, des registres de dispensation d'ARV et des fiches de stock des pharmacies des structures sanitaires visitées. L'extraction des données pour les IAP, a été faite au niveau de chaque site par un agent formé par l'équipe de coordination, aidé par le gestionnaire de données du site, à l'aide de fiches de collecte de données élaborées par la coordination. Les fiches renseignées et validées par un superviseur, ont été ensuite acheminées au niveau central. La saisie des données a été faite dans l'outil d'extraction des IAP développé par l'OMS (version mai 2010) [8]. L'analyse des données a été faite à partir de ce même outil, par les membres du groupe technique de travail pour la surveillance épidémiologique du VIH. Les valeurs de chaque indicateur sont calculées directement par la base de données, pour chaque site. Quatre Indicateurs d'Alerte Précoce (IAP) ont été retenus sur les 8 que propose l'OMS. Ce choix se justifie par le fait qu'après l'analyse situationnelle, seuls ces quatre indicateurs pouvaient être renseignés. Il s'agit de: (i) pratiques de prescription du TARV (IAP1 pour adultes et ou IAP1-P pour enfants), (ii) patients perdus de vue au cours des 12 premiers mois de TARV (IAP2), (iii) patients sous schéma thérapeutique de TARV de première intention approprié à 12 mois (IAP3a ou IAP3a-P), (iv) constance de la délivrance des ARV (IAP6a). Les objectifs nationaux pour les indicateurs d'alerte précoce de la résistance du VIH aux ARV sont ceux proposés par l'OMS, c´est-à-dire IAP1=100%, IAP2 ≤20%, IAP3 ≥70% et IAP6=100% [4, 9].

## Résultats

**Figure 1:**
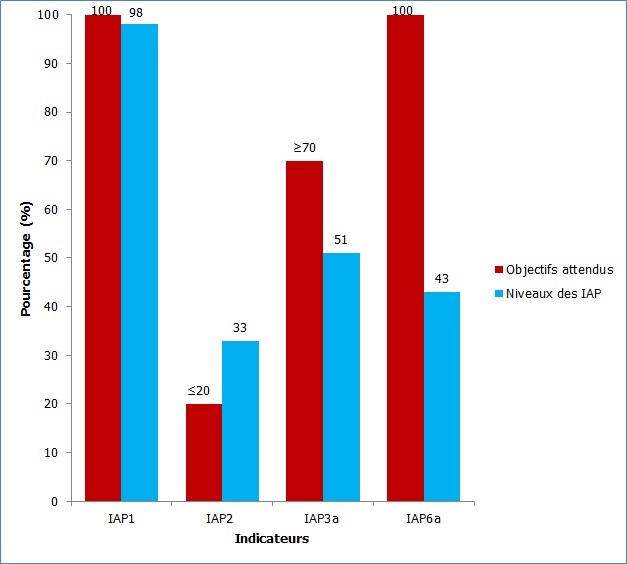
Objectifs attendus et niveaux des IAP sur l’ensemble des 20 sites de TARV en 2008-2009 en Côte d’Ivoire

**Caractéristiques générales de l'échantillon et niveau des indicateurs sur l'ensemble des sites de TARV:** Au total 1919 PVVIH ont été enrôlées, dont 1795 adultes provenant de 18 sites et 124 enfants de 2 sites. Le niveau de chaque indicateur sur l'ensemble des sites, comparé à l'objectif attendu est représenté par la Figure 1.

**Niveau des indicateurs par site de TARV. *Pratiques de prescription du TARV :*** Pour cet indicateur, 4 sites sur 20 enquêtés (20%) ont atteint l'objectif de 100% de prescriptions initiales conformes aux directives nationales. Onze sites (55%) avaient 98% à 99% de prescriptions initiales conformes et 5 sites (25%) en avaient 92% à 97%. Les sites qui avaient moins de 97% de prescriptions initiales conformes étaient au nombre de 2 soit 10% (Tableau 1).

**Tableau 1:** Synthèse des indicateurs d’alerte précoce de la résistance du VIH aux ARV sur 20 sites de TARV en 2008-2009 en Côte d’Ivoire

Sites de TARV	IAP1, IAP1-P: pourcentage de prescriptions de schéma thérapeutique de TARV initial approprié (%)	IAP2: pourcentage de patients initiant un TARV de première intention et perdus de vue 12 mois plus tard (%)	IAP3a: Pourcentage de patients sous schémas thérapeutique de TARV de première intention approprie à 12 mois (%)	IAP6a: pourcentage de mois sans aucune rupture de stocks d'ARV au cours d’une année (%)
**Oblectifs**	100 %	≤20 %	≥70 %	100 %
CAT Adjamé	98 (196/199)	ND	ND	50 (6/12)
Centre Nazaréen Yopougon	98 (98/100)	6 (6/97)	86 (85/99)	67 (8/12)
CEPREF Yopougon	98 (48/49)	16 (7/43)	73 (35/48)	0 (0/12)
CHR Agboville	99 (74/75)	48 (34/71)	33 (24/72)	42 (5/12)
Espace Confiance Zone 4	99 (99/100)	24 (22/90)	67 (60/89)	8 (1/12)
FSUCOM Wassakara Yopougon	97 (119/123)	42 (45/108)	45 (47/105)	25 (3/12)
HG Abobo	98 (126/129)	40 (46/114)	44 (51/115)	33 (4/12)
HG Adzopé	99 (109/110)	32 (29/90)	47 (47/101)	50 (6/12)
HG Alépé	97 (97/100)	37 (30/82)	50 (45/90)	58 (7/12)
HG Anyama	97 (95/98)	43 (40/92)	41 (38/93)	75 (9/12)
HG Ayamé	98 (107/109)	42 (28/86)	43 (32/74)	42 (5/12)
HG Bonoua	93 (102/110)	62 (53/86)	22 (20/91)	ND
HG Grand Bassam	99 (102/103)	35 (31/88)	65 (55/84)	ND
HG Grand Lahou	98 (45/46)	28 (11/39)	50 (21/42)	25 (3/12)
HG Sikensi	100 (29/29)	18 (3/17)	50 (11/22)	100 (12/12)
HG Toumodi	100 (93/93)	23 (12/52)	35 (28/80)	50 (6/12)
Hôpital Protestant Dabou	98 (115/117)	32 (37/114)	52 (60/115)	50 (6/12)
Neurologie CHU Cocody	92 (22/24)	41 (7/17)	43 (9/21)	42 (5/12)
Pédiatrie CHU Treichville	100 (75/75)	12 (8/67)	66 (48/73)	17 (2/12)
PPH CHU Cocody	100 (130/130)	33 (42/127)	61 (77/126)	42 (5/12)

***Patients perdus de vue au cours des 12 premiers mois de TARV :*** Un an après le début du traitement, 4 sites sur 19 (21%) ont atteint l'objectif de moins de 20% de patients perdus de vue. Quatorze sites (74%) avaient une proportion de perdus de vue allant de 23% à 48%. Un site (5%) avait 62% de perdus de vue (Tableau 1).

***Patients sous schéma thérapeutique de TARV de première intention approprié à 12 mois :*** Sur 19 sites pour lesquels cet indicateur a pu être calculé, 2 sites (11 %) ont atteint l'objectif d'au moins 70% de patients sous schéma thérapeutique de TARV de première intention approprié à 12 mois. Pour 8 sites (42%), cette proportion allait de 50% à 67% et pour 9 sites (47%), de 22% à 47% (Tableau 1).

***Constance de la délivrance des ARV :*** Sur 18 sites pour lesquels cet indicateur a été calculé, 1 site (6%) n'a connu aucune rupture d'ARV au cours des 12 mois de l'étude. Des ruptures de stock d'au moins un ARV allant de 1 à 9 mois ont été constatées sur 16 sites. Une rupture de stock d'au moins un ARV a été constatée sur 12 mois (Tableau 1).

## Discussion

***Pratiques de prescription du TARV:*** Les bonnes pratiques de prescription du TAR n'étaient pas toujours observées par tous les prescripteurs sur la majorité des sites. En effet, seulement 20% des sites ont atteint l'objectif de 100% de prescription initiale conformes aux directives nationales. Cela traduirait une appropriation insuffisante et le non respect des directives nationales de prescription du TARV par certains praticiens en 2008. En effet, après la formation des prescripteurs, la mise en œuvre du coaching et du suivi post formation n'était pas toujours effective. Cependant, on remarque que 75% des sites avaient au moins 98% de prescriptions initiales conformes aux directives nationales et que seulement 10% des sites avaient un taux de prescriptions initiales conformes en dessous de 97%. L'enquête réalisée en 2009 en Côte d'Ivoire par l'ONG ACONDA-VS, a montré que 10 sites sur 16 soit 62,5% avaient atteint l'objectif de 100% [10]. Les évaluations réalisées au Togo en 2009 et au Cameroun en 2011 montraient des résultats plus satisfaisants avec respectivement 100% [11] et 90% [12] des sites qui ont atteint l'objectif. Au Togo, la bonne performance pourrait s'expliquer par deux faits : d'une part la validation de toutes les premières prescriptions par les comités thérapeutiques sur la base des protocoles ARV, d'autre part la vérification des ordonnances sur les sites de dispensation des ARV par la centrale d'achat des médicaments génériques et ses dépôts régionaux [11]. Pour le Cameroun, la performance atteinte serait due à la prescription des traitements lors des réunions hebdomadaires des comités thérapeutique dans les centres de traitement agréés [12].

***Patients perdus de vue au cours des 12 premiers mois de TARV:*** Le taux de patients perdus de vue au cours de leur première année de traitement est élevé sur l'ensemble des sites (33%) et peu de sites (4/19) ont atteint l'objectif de moins de 20% de perdus de vue. L'enquête pilote réalisée en 2008 à Abidjan montrait également que très peu se sites (2/14) avaient atteint cet objectif [12]. L'importante proportion de perdus de vue pourrait traduire une insuffisance du suivi des patients sous TARV dans la majorité des sites enquêtés. Cette situation s'expliquerait par l'inexistence d'un système national formel de rappel et de recherche des patients absents à leur rendez-vous. Elle traduirait également une insuffisance d'engagement du patient à suivre et à respecter son traitement, l'absence de ressources financières pour le suivi des patients absents à leur rendez-vous ou une sous notification des rendez-vous aux patients et dans leur dossier.

***Patients sous schéma thérapeutique de TARV de première intention approprié à 12 mois :*** La moitié (51%) des patients mis sous un TARV de première intention demeurait sous un schéma thérapeutique de première intention appropriée 12 mois plus tard sur l'ensemble des sites; ce qui est en deçà de l'objectif d'au moins 70%. Ceci pourrait être la conséquence de plusieurs situations : (i) le taux élevé de patients perdus de vue avant la fin de la première année de traitement, (ii) certains patients ne disposant pas d'ARV à 12 mois en raison du non respect des rendez-vous ou en raison de rupture de stock à la pharmacie, (iii) la prescription ou le retrait d'un schéma thérapeutique de première intention pas toujours conforme aux protocoles thérapeutiques en vigueur ou (iv) un échec thérapeutique de la première intention entrainant un changement de prescription.

***Constance de la délivrance des ARV :*** Les ruptures en ARV étaient fréquentes au cours de la période de l'étude (1 seul site n'a pas connu de rupture). Cette fréquente rupture a sans doute contribué au faible taux de patients sous schéma thérapeutique de TARV de première intention approprié à 12 mois. La faible constance de la délivrance des ARV a pu être provoquée par une mauvaise gestion des stocks sur les sites ou par une insuffisance d'approvisionnement des produits par les structures de distribution. Cependant certaines ruptures d'ARV n'empêchaient pas toujours la délivrance du traitement aux patients du fait de la possibilité de substitution.

## Conclusion

Des insuffisances dans la prise en charge par les ARV des PVVIH ont été relevées sur la période de 2008 à 2009. Ces insuffisances objectivées par le niveau des indicateurs évalués, supposent l'existence de risque important de survenu de résistance du VIH aux ARV. En effet, si la plupart des prescripteurs s'étaient approprié et observaient les pratiques de prescription des ARV conformément aux directives nationales, des efforts restaient à faire chez certains praticiens. L'amélioration des pratiques de prescription pourrait se faire grâce à des prescriptions collégiales et des supervisions formatives. La mise en place d'un système formel de rappel et de recherche active des absents aux rendez-vous s'avère nécessaire pour minimiser l'importance des perdus de vue. Les structures d'approvisionnement et les sites de TARV devraient tout mettre en œuvre pour assurer une disponibilité constante des ARV sur les sites. Tous ces efforts devraient contribuer de façon significative à minimiser les risques de survenu de résistance du VIH aux ARV.

### Etat des connaissances actuelles sur le sujet

- Ces indicateurs sont importants pour évaluer le risque de survenu des résistances du VIH aux antirétroviraux.
- Le niveau de ces indicateurs est très variable dans les premières études et selon les pays.

### Contribution de notre étude à la connaissance

- Cette étude a un intérêt en santé publique, spécifiquement dans le domaine du VIH. Ce travail devrait pouvoir aider à comprendre les risques de survenue de résistance du VIH aux ARV qui sont liés à des défaillances dans la prise en charge de la maladie par les ARV surtout dans nos pays en développement.
- Il devrait donc pouvoir aider les acteurs de la lutte contre le VIH à surveiller et améliorer les indicateurs d'alerte précoce pour réduire les risques de résistance.
- Pour la Côte d'Ivoire, ce travail permet de savoir le niveau de ces indicateurs sur la période de l'étude et permettra de voir l'évolution de ces indicateurs, avec les prochaines études qui seront faites.
